# Mimicking Pathogens to Augment the Potency of Liposomal Cancer Vaccines

**DOI:** 10.3390/pharmaceutics13070954

**Published:** 2021-06-24

**Authors:** Maarten K. Nijen Twilhaar, Lucas Czentner, Cornelus F. van Nostrum, Gert Storm, Joke M. M. den Haan

**Affiliations:** 1 Department of Molecular Cell Biology and Immunology, Cancer Center Amsterdam, Amsterdam Infection and Immunity Institute, Amsterdam University Medical Center, Vrije Universiteit Amsterdam, 1081 HZ Amsterdam, The Netherlands; m.nijentwilhaar@amsterdamumc.nl; 2 Department of Pharmaceutics, Faculty of Science, Utrecht University, Universiteitsweg 99, 3584 CG Utrecht, The Netherlands; l.czentnercolomo@uu.nl (L.C.); C.F.vanNostrum@uu.nl (C.F.v.N.); G.Storm@uu.nl (G.S.); 3 Department of Biomaterials, Science and Technology, Faculty of Science and Technology, University of Twente, 7522 NB Enschede, The Netherlands; 4 Department of Surgery, Yong Loo Lin School of Medicine, National University of Singapore, Singapore 119228, Singapore

**Keywords:** liposomes, cancer vaccines, antigen-presenting cells, targeting, pathogen mimicry

## Abstract

Liposomes have emerged as interesting vehicles in cancer vaccination strategies as their composition enables the inclusion of both hydrophilic and hydrophobic antigens and adjuvants. In addition, liposomes can be decorated with targeting moieties to further resemble pathogenic particles that allow for better engagement with the immune system. However, so far liposomal cancer vaccines have not yet reached their full potential in the clinic. In this review, we summarize recent preclinical studies on liposomal cancer vaccines. We describe the basic ingredients for liposomal cancer vaccines, tumor antigens, and adjuvants, and how their combined inclusion together with targeting moieties potentially derived from pathogens can enhance vaccine immunogenicity. We discuss newly identified antigen-presenting cells in humans and mice that pose as promising targets for cancer vaccines. The lessons learned from these preclinical studies can be applied to enhance the efficacy of liposomal cancer vaccination in the clinic.

## 1. Introduction

Liposomes are versatile drug delivery vehicles because of their ability to transfer both hydrophobic and hydrophilic substances. In addition, liposomes are known for their low immunogenicity, a good safety profile, and their biocompatibility. Hence, interest in liposomal delivery vehicles has emerged in various fields [1]. In the field of oncology, the last decades have been marked by major developments in combating cancer cells with chemotherapy or molecular targeted therapies (e.g., targeting of the proteasome, receptors for growth factors) [2]. However, the systemic (soluble) application of these therapies can have severe side effects, thereby limiting the therapeutic window [2]. In order to facilitate a more effective and safe therapy, liposomes then emerged as vehicles to directly deliver these drugs to cancer cells [3]. Furthermore, liposomes gained interest because of their potential use in modulating the tumor microenvironment by direct delivery of immunomodulatory drugs to tumors. Indeed, liposomes can enter tumors because of the enhanced permeability and retention effect caused by the relatively permeable (“leaky”) tumor vasculature [4]. Simultaneously, major developments in the field of immunology were made to engage the immune system against cancer [5,6]. As cancers gain mutations and start to aberrantly overexpress tissue-restricted proteins during malignant transformation, they are recognized by the immune system as “non-self”. Hence, similar to infectious agents, this opens a therapeutic window to harness the immune system in the fight against “non-self” [7]. Indeed, immunotherapies that reinvigorate the exhausted anti-cancer immune response (e.g., immune checkpoint blockade) have been successful in a limited number of cancers and patients, and it is envisaged that this could be enhanced by means of vaccination [8,9,10].

In contrast to vaccinations against infectious agents that require strong induction of (neutralizing) antibody responses, cancer vaccination strategies are more focused toward induction of cytotoxic CD8^+^ T cell responses that are capable of tumor cell killing [11]. This indicates that direct translation of methods used in vaccines against infectious diseases may be less effective in cancer vaccination. In addition, due to the low immunogenicity of cancer antigens, vaccine effectiveness is often suboptimal, pointing to the need to improve the potency of vaccine technologies [12]. Hence, liposomes, and also other nanoparticulate carriers, received great interest in immune-oncology as carriers of antigens and adjuvants. Optimized cancer vaccines in combination with immune checkpoint blockade can potentially synergistically induce antitumor immunity by eliciting highly effective de novo or reinvigorated T cell responses and, consequently, improved clinical responses [5,6,13].

In this review, we aim to provide an overview of preclinical studies that address the efficacy of liposomal cancer vaccines and relate it to immunological processes important for T cell priming. Although there is a wide variety of nanoparticulate carriers that can be used in vaccination strategies, we have limited ourselves to liposomes and occasionally include related lipoplexes, as the latter lipid-based carriers are often used and particularly designed for the delivery of nucleic acids encoding for antigens. In this review, we will first discuss the characteristics of the two major cancer vaccine components, antigen and adjuvant, and how the incorporation of these two components into liposomes enhances their immunogenicity or potency, respectively. Secondly, we discuss how the different administration routes and biochemical characteristics of liposomes influence uptake in secondary lymphoid organs, presentation by antigen-presenting cells (APCs) and T cell priming. We provide an overview of different types of APCs, including newly identified subsets, and discuss their functions in T cell priming. Finally, we describe how the incorporation of targeting moieties derived from pathogens leads to more efficient uptake by specific APCs and further enhances the immunogenicity of vaccines. In summary, liposomes that incorporate antigen, stimulatory adjuvant, and targeting moieties start to resemble pathogens, and this pathogen mimicry may provide perspectives for the improvement of liposomal cancer vaccines in the near future.

## 2. Antigen and Adjuvant Are the Basic Ingredients of Vaccines

### 2.1. Classification of Tumor Antigens

The immune system recognizes foreign antigens and can react to these molecules that are present in pathogens, infected, or malignantly transformed cells. One class of antigens that is present in cancer cells is normal proteins that are aberrantly expressed or overexpressed. These antigens are normally restricted to distinct tissues such as the testis or are only expressed at low levels and are termed cancer testis antigens (CTAs) and tumor associated antigens (TAAs). These antigens can be immunogenic and are potential cancer antigens that can be used for vaccination strategies [12]. Since these antigens are shared by multiple patients, they present as possible targets for “off the shelf” vaccination strategies. However, CTAs and TAAs are normal proteins and can therefore be classified as “self”. Tolerance to self-antigens is achieved in the thymus, in which T cells with a high affinity T cell receptor (TCR) for self-antigens are deleted. As a consequence, remaining T cells that may react to these antigens have a low affinity TCR, which may hamper their effectiveness. This is especially relevant for TAA-specific T cells, as tolerance induction for CTA-specific T cells is less stringent [12].

In addition to CTAs and TAAs, tumor cells are marked by a high mutational load that leads to an accumulation of proteins with mutations that are termed as neoantigens or tumor-specific antigens [14]. These mutations are highly specific for a patient or a model used and can be identified by means of high-throughput screening methods (e.g., next generation sequencing). Since no tolerance to newly developed mutations exist, neoantigens are considered to be highly immunogenic and can elicit responses from T cells with a high affinity TCR [15]. Thus, cancer antigens can be broadly divided in aberrant or overexpressed proteins shared by multiple patients and newly developed mutations that are highly patient-specific, and both types of antigens can be included in cancer vaccines.

### 2.2. Vaccine Antigen Can Be Incorporated in Various Forms

While CD8^+^ cytotoxic T cells recognize peptides in the context of major histocompatibility complex (MHC), vaccination strategies can make use of different forms of the antigen to prime the immune system, such as peptides, protein, DNA, or RNA. Initially, short peptides that represent viral epitopes were used to vaccinate against virally induced cancers [16]. However, short peptides were subsequently found to be prone to tolerance induction and, instead, longer peptides that do need processing, like protein antigen, were preferred [16].

In addition to long peptides and protein antigen, more recently major developments in nucleotide vaccines have been made. This is highlighted by the recent effectiveness of mRNA vaccines against COVID-19 [17]. This vaccination platform emerged from a longer standing interest in mRNA vaccines to treat cancer [18]. Indeed, an mRNA-based vaccination platform was used for cancer patients, where mRNA encoded multiple neoantigens that were present in their tumors [19]. In addition, mRNA vaccines can also be used to encode multiple TAAs [20,21]. The former possibility opens a window to personalized cancer vaccines and the latter one to “off the shelf” cancer vaccines. In order to augment the potency of these vaccines, mRNA that encodes CD4^+^ helper T cell epitopes may also be included in the vaccine formulation [22].

In comparison to peptide and/or protein antigen, mRNA molecules are known to be relatively instable, which may hamper their effectiveness. In order to overcome this hurdle, multiple modifications to the 5′-cap, poly(A) tail and 5′ and 3′ untranslated regions can be made to make the mRNA more stable and effective. These alterations enhance the magnitude of the response (translation efficiency) as well as the half-life of mRNA (duration of the response) [18]. Another potential drawback of these vaccines is the need for uptake of relatively large negatively charged mRNA molecules by cells, which is generally considered to be relatively inefficient. However, especially immature dendritic cells (DCs) are able to take up naked mRNA molecules, albeit to a low extent. Importantly, vaccination with naked mRNA successfully resulted in the priming of antigen-specific T cell responses upon intradermal or intranodal administration [23,24]. In order to enhance the stability and/or uptake of these vaccines, mRNA is often complexed with lipids to create lipoplexes, which can further augment vaccine effectiveness [20,21].

### 2.3. Cancer Vaccination Aims to Induce CD8^+^ T Cell Responses via Cross-Presenting DCs

In order to mount a cytotoxic T cell response specific to cancer cells, CD8^+^ T cells should be activated by DCs that present cancer antigens [25]. DCs can take up cancer antigens at the tumor site or in the draining lymph node (LN). Subsequently, DCs process and present these antigens in their MHC molecules, MHC class I or II to elicit responses by CD8^+^ or CD4^+^ T cells, respectively [26,27]. MHC class I is present on all nucleated cells and is usually loaded with endogenous peptides derived from the cytosol. However, DCs have the unique capacity to cross-present peptides in MHC class I. Here, peptides are derived from exogenous antigens that have been phagocytosed, such as dying cancer cells. This latter process is called cross-presentation and is restricted to DCs. However, different types of DCs exist and not all DCs have the same capacity for cross-presentation (further described in “Different APCs for T cell priming”) [28].

Two major routes have been described for cross-presentation, the cytosolic and vacuolar route [29,30]. Cross-presentation via the cytosolic pathway requires antigen translocation to the cytosol, proteasomal degradation of antigen, and subsequent TAP-dependent transport to the endoplasmic reticulum (ER) or back to the phagosome. Hence, antigen should escape, or be released, from phagosomal compartments to gain access to the cytosol. Although the mechanism of transport is under debate, recent work has shown that phagosomal rupture is a potential mechanism [31]. In contrast to the cytosolic route, cross-presentation via the vacuolar route occurs fully in endo-phagosomal compartments and is not dependent on cytosolic proteases, but is sensitive to inhibition of lysosomal proteolysis [26,32]. Interestingly, the mechanism of vaccine uptake influences antigen routing and subsequent cross-presentation (further described in “Active targeting leads to uptake by APCs of choice”) [33,34]. Thus, CD8^+^ T cell responses are elicited by cross-presenting DCs, and the efficiency of this process is largely determined by the targeted DC subtype and its capacity of cross-present.

### 2.4. Vaccine Adjuvants Are Essential to Induce an Immune Response

In addition to antigen, cancer vaccines should be supplemented with an adjuvant to mature DCs, which can subsequently provide essential costimulatory signals to naïve T cells during their priming. Adjuvant-induced DC maturation enhances the density of MHC–peptide complexes on the cell surface, but also stimulates expression of costimulatory markers such as CD40, CD80, and CD86, while simultaneously triggering cytokine secretion [35]. The maturation of DCs can be triggered by bacterial or viral infections, and cancer vaccines can include bacterial or viral components as adjuvant. These bacterial or viral components consist of pathogen-associated molecular patterns (PAMPs) that can be recognized by pattern recognition receptors, such as a toll-like receptors (TLRs), present on DCs [36,37]. TLRs can be found on the cell surface, but can also reside in endosomal compartments, dependent on the nature and localization of their ligand [36,37]. Importantly, TLRs are not ubiquitously expressed by all DC types [38]. In addition to TLRs, innate immune receptors in the cytosol, such as RIG-I, MDA-5, and STING, may be activated to provide immune activation [39,40,41]. When a cancer vaccine contains nucleotides (i.e., DNA or RNA) the vaccine may not require additional TLR ligands or other activation signals due to the intrinsic adjuvant effect caused by binding of nucleotides to TLRs [20].

Interestingly, next to the effects of adjuvant on DC maturation and costimulatory capacity, TLR stimulation was also found to promote fusion of phagosomes (containing exogenous antigen) with endosomal recycling compartments (containing MHC class I molecules). Thereby, exogenous antigen is brought in close proximity to MHC class I-containing compartments, which promotes antigen cross-presentation [42,43,44]. As this route of antigen loading is distinct from endogenous antigen loading on MHC class I (in the endoplasmatic reticulum), this points to a preferential stimulation of the cross-presentation route upon TLR stimulation [45].

In conclusion, two major prerequisites for successful cancer vaccines are inclusion of an antigen, to which the subsequent immune response should be directed, and an immune stimulating agent, adjuvant, to ensure proper DC maturation. Importantly, since many cell types can potentially take up vaccine components but cannot induce T cell responses, specific targeting of DC subsets with both vaccine antigen and adjuvant, taking heterogeneous expression of TLR and antigen processing capacity into account, can increase vaccine potency.

## 3. Liposomes as Delivery System

### 3.1. Liposomal Antigen Accumulation in Lymph Nodes

Since CD8^+^ T cell activation by DCs can only occur in secondary lymphoid organs (LN) or spleen, antigens that are administered in other tissues should be transported to these lymphoid organs. After antigen injection via conventional routes (e.g., intradermal (i.d.), intramuscular (i.m.), or subcutaneous (s.c.)), antigen can accumulate in the LN via local uptake at the site of injection by tissue-resident APCs that subsequently migrate to the LN. Alternatively, antigen itself may passively drain from the site of injection via the lymphatic system to the LN. The potential use of liposomes and their physicochemical properties largely determine how and where the immune system encounters antigen (Table 1). For example, the majority of freely administered (i.e., not encapsulated) protein antigen was found to drain from the site of injection within a day, while encapsulation of the antigen in cationic liposomes resulted in electrostatic interactions with the tissue and the retention of antigen at the site of injection (i.m.) over multiple days [46]. In fact, cationic liposomes appear to serve as a depot from which antigen is slowly released over time [46]. In contrast, neutral and anionic liposomes drain more easily upon i.m. injection to the LN [47,48]. The sustained release characteristics of a liposomal antigen depot are dependent on the charge, membrane fluidity, and retention time of the liposomes at the injection site [49]. In addition, direct interaction of the liposomes with tissue-resident APCs and subsequent internalization by these cells can also be an important determining factor [50]. Thus, the degree of liposome uptake is dependent on surface charge and membrane fluidity as they influence the interaction with the extracellular matrix and cells.

Surface stabilization of liposomes with polymers (e.g., polyethylene glycerol (PEG)) can result in enhanced draining of the liposome particles from the site of injection [51]. While relatively low mole percentages of PEG (1 mol %) were indeed found to improve draining to the regional lymphatics and subsequent localization in the LN, incorporation of a higher quantity of PEG (5 mol %) facilitated lymphatic drainage, but simultaneously hindered antigen accumulation in the draining LN due to lower antigen uptake by APCs [52]. PEG can also shield phospholipids in liposomal preparations, so that specific recognition of, for example, phosphatidylserine (PS), that is normally present on apoptotic cells and is a ligand for APCs, is hindered [47,53]. Similarly, PEG can shield specific targeting moieties on the liposome surface, and therefore liposomal stabilization with PEG may have an unfavorable effect on targeting capacity [54]. However, coupling of targeting ligands to the terminal ends of PEG can be utilized to overcome this [55]. Of note, liposomal surface decoration with PEG also makes the liposomes immunogenic [56]. Thus, the altered targeting efficiency and immunogenicity indicate that addition of PEG to liposomal preparations should be carefully assessed.

The size of liposome particles further influences the capacity of liposomes to drain to the LN as particles that were smaller than 150 nm drained to the regional lymphatics while larger particles stayed at the injection site after an s.c. injection [57,58]. Upon i.m. injection, smaller particles (100 nm) also drained rapidly, while larger particles (900 nm) were retained [47]. Thus, smaller particles tend to rapidly drain, while relatively large particles are not well equipped to diffuse to the lymphatic system and their localization in the LN is dependent on peripheral uptake and subsequent migration of tissue-resident immune cells [59].

**Table 1:** Physicochemical properties of liposomes determine liposomal behavior upon injection.

Size	Charge	Administration Route	Effect	References
~100 nm	Cationic	i.m.	Depot formation	[46]
~100 nm	Neutral and anionic	i.m.	Draining via the regional lymphatics to the LN	[47]
‘Large’ 500–900 nm	Cationic	s.c.	Retention at the site of injection	[47,58]
‘Small’ 100–140 nm	Cationic	s.c.	Enhanced draining to the LN as compared to larger counterparts	[47,58]
~250 nm	Cationic	s.c.	Liposomal surface decoration with PEG enhanced draining to the LN	[52]
~300 nm	Neutral or anionic	i.v.	Antigen uptake in the spleen	[20]
~160 nm	Cationic	i.v.	Antigen sequestration in the lung	[60]

Thus, the rate and extent of liposome drainage from the site of injection into the lymphatics are dependent on their physicochemical properties. In general, a cationic nature or large size of liposomes results in strong retention at the injection site and subsequent release of antigen (depot formation), while small, PEGylated, anionic, or neutral liposomes drain more easily to the LN (Table 1).

### 3.2. Liposomal Antigen Uptake in the Spleen

When liposomes are injected intravenously (i.v.), uptake or clearance of liposomes is mostly performed by phagocytic cells of the mononuclear phagocytic system (MPS) present in spleen and liver [61,62]. As the spleen is a lymphoid organ that mediates immune responses, i.v. administration is a potential route for vaccines to facilitate T cell priming [20,21,63]. Similar to other administration routes, physicochemical characteristics of the liposomes play a major role in their in vivo behavior. Hence, modifications of the surface of liposomes may be used to optimize splenic targeting. Indeed, i.v. administration of neutral or negatively charged lipoplexes resulted in considerable antigen uptake in the spleen [20]. However, administration of positively charged particles resulted in predominant uptake in the lung and, to a lesser extent, in the spleen and liver (Table 1) [60]. This difference in biodistribution between cationic and anionic lipoplexes was corroborated in a side-by-side comparison where cationic particles were found to predominantly accumulate in the lung and anionic particles in the spleen and liver [20,64]. Particle sequestration in the lung can be explained by rapid aggregation in the bloodstream induced by the interaction of cationic particles with circulating blood proteins and cells. This can result in rapid clearance from the bloodstream and can have serious side effects [65]. Importantly, the sequestration of these positively charged particles in the lung is likely dependent on the extent to which lipoplexes interact with blood-borne molecules and cells. When lipoplexes composed of different cationic lipids were assessed for their biodistribution, differences in the relative distribution over multiple organs (lungs, liver, spleen, and kidney) were found, indicating that sequestration is not solely dependent on charge, but also on the lipids used to complex with the nucleotides [66].

Thus, the physicochemical characteristics and the biodistribution profile of cancer vaccines that are intravenously administered should be carefully assessed and tailored to optimally induce a robust immune response.

### 3.3. The Route of Vaccine Administration Dictates Subsequent Responses

While the physicochemical characteristics of liposomes affect their subsequent interaction with tissues after immunization, alterations in the immunization route can also impact the quality and magnitude of the subsequent responses (Table 2) [67]. When cationic liposomes were evaluated, i.d. injection elicited superior anti-tumor responses as compared to s.c. immunization [68]. We observed a lower magnitude of the immune response upon s.c. immunization versus i.v. immunization when we evaluated anionic liposomes [54].

Although i.v. immunization is considered to be rather unconventional, i.v. administration of mRNA lipoplexes made it to the clinic and have been tested in patients with melanoma [20,21,63]. Lipoplexes were also tested for their effectiveness upon i.d., i.m., or s.c. administration. Intriguingly, the effectiveness of immunization with lipoplexes was found to be severely hampered upon s.c. or i.d. administration [69], which was in sharp contrast to the effectiveness of i.v. administration [20,21]. When the i.v. administration route was directly compared to the i.m./s.c. or to the i.d./s.c. route, respectively, both of these studies showed superior responses when lipoplexes were administered i.v. [70,71]. Intriguingly, s.c. delivery of lipoplexes was associated with release of type I interferon (IFN) that significantly hampered priming of cytotoxic T cells [69]. Conversely, the potent effect of i.v. administered lipoplexes was abrogated by blocking type I IFN signaling [71]. Hypothetically, sensing of either mRNA or IFN results in a cellular response that is accompanied by a lower translational activity, and thereby reduces the amount of protein antigen obtained with mRNA vaccines. In contrast, type I IFN has also been shown to promote DC maturation and to induce anti-cancer T cell responses [72,73]. In an attempt to shed light on these observations, van Hoecke and co-workers observed that a selective knockout (KO) of the type I IFN receptor (IFNR) on T cells improves immune responses upon s.c. administration of lipoplexes, but reduces T cell responses upon i.v. administration of lipoplexes [74]. In contrast to a selective IFNR-KO on T cells, a selective IFNR-KO on DCs was found to be of less importance [74]. This surprising finding indicates that the route of immunization has a major role in the potency of vaccination and that similar factors have a different role dependent on the route of immunization.

To conclude, these findings stress that successful vaccination with liposomes and lipoplexes is highly dependent on the route of administration and this should be carefully chosen on the basis of experimental in vivo studies.

### 3.4. Antigen Encapsulation Augments Antigen Immunogenicity

Uptake of soluble administered antigen is not solely restricted to professional APCs, which can hamper vaccine effectiveness, because steady-state scavenging non-professional APCs, such as liver sinusoid endothelial cells and lymphatic endothelial cells, are capable of antigen processing and presentation in MHC class I, leading to tolerance induction [75,76]. Furthermore, immunization with soluble minimal peptides that do not need processing also led to tolerance induction after an initial phase of activation. These minimal peptides directly bound to MHC class I molecules on non-activated cells that subsequently induced tolerance [16]. Therefore, cancer vaccine formulations should prevent erroneous off-targeting and use of soluble minimal peptides that may be taken up by non-professional APCs.

As previously mentioned, dependent on the size and lipid composition, liposomal encapsulation of antigen can lead to depot formation at the site of injection. Indeed, radioactively labeled antigen and liposomes were retained at the site of injection upon i.m. injection, in sharp contrast to antigen that was injected without liposomes and that was quickly cleared [77]. Interestingly, the difference in antigen retention also translated to differences in the magnitude of the immune response, as CD8^+^ T cell responses were observed when antigen was injected with liposomes, but not in absence of liposomes [77]. Indeed, because of the endured antigen accessibility and slower release characteristics, the immune reaction, as determined by CD8^+^ T cell responses, can increase in potency compared to administration of free peptide (Table 2) [78,79]. DCs were also shown to more efficiently present liposomal-associated peptide compared to soluble peptide [80,81]. When we assessed the peptide immunogenicity in vivo, we observed that incorporation of peptide in liposomes dramatically augmented the immunogenicity. While immunization with liposomal peptide resulted in potent CD8^+^ T cell responses, an equal dose of soluble antigen failed to elicit a noticeable immune response [54]. In agreement with these findings, encapsulation of peptide in liposomes was found to be an absolute requirement for anti-tumor activity [82]. In addition to peptide antigen, the immunogenicity of protein antigen was also found to be enhanced by incorporation in liposomes [83].

While the main focus in cancer vaccination is on CD8^+^ T cells, the role of CD4^+^ T cells should not be overlooked [84]. We observed that the priming of CD4^+^ T cells was also markedly enhanced upon immunization with liposomal, rather than soluble, peptide [54]. In an elaborate study, Varypataki and co-workers corroborated the importance of CD4^+^ T cell epitope inclusion and peptide encapsulation. They made use of OVA-derived synthetic long peptides (SLPs) containing either a CD8^+^ or a CD4^+^ T cell epitope. Immunization with mixed-in soluble SLPs and poly(I:C) as adjuvant of choice did not elicit a noticeable immune response. In contrast, when these SLPs with a CD8^+^ or CD4^+^ T cell epitope were loaded into liposomes the induction of antigen-specific immune responses upon immunization was markedly increased. In two different tumor models, enhanced immune responses and a delayed tumor growth were observed when antigens were administered in a liposomal context, further supporting the notion that antigen encapsulation is of importance for vaccine effectiveness [68].

Similar to protein or peptide antigen, formation of lipoplexes increased the potency of nucleotides as antigen in vitro [85]. While this is important for protection of nucleotide integrity, the uptake of RNA lipoplexes was also found to be enhanced [85,86].

Thus, for different types of antigen, liposomal encapsulation can facilitate slower antigen release and promote cellular antigen uptake, which leads to enhanced immune responses (Table 2).

**Table 2:** Liposomal encapsulation of antigen and adjuvant as well as surface modification with targeting moieties augments vaccine potency in vivo.

Administration Route	Antigen	Adjuvant	Targeting (Moiety)	Targeted or Activated Cell	Effect	References
i.p.	OVA protein	CpG	Passive	N/A	Enhanced responses compared to soluble vaccine components	[87]
i.p.	OVA protein	CpG or Poly IC	Passive	DC and macrophage	Enhanced responses upon combination of TLR ligands	[88]
i.d.	OVA protein	CpG	Passive	N/A	Enhanced responses compared to soluble vaccine components	[89]
Intratumoral	N/A	aCD40 and CpG	Antibody mediated	Intratumoral macrophage and DC	Enhanced responses compared to soluble vaccine components	[90]
Inhalation	N/A	Cyclic dinucleotides	PS	DC and macrophage	Synergized anti-tumor responses when combined with irradiation	[91]
Hypodermic (not specified)	Melanoma peptides	CpG	Mannose	DC	Enhanced responses compared to soluble vaccine components	[92]
s.c.	OVA peptide	Poly IC and Pam3CSK_4_	Passive	N/A	Enhanced responses compared to soluble peptide	[78]
s.c.	OVA protein	Cyclic dinucleotides	Passive	DC and macrophage	Enhanced responses compared to soluble vaccine adjuvant	[93]
s.c.	Human papilloma virus peptide	CpG	Mannose	DC	Enhanced responses compared to soluble vaccine components	[82]
s.c.	Melanoma antigen mRNA	N/A	mannose-mimicking head group	DC	Protection from tumor challenge	[94]
s.c.	ErbB2 protein	Pam(3)CAG, Pam(2)CAG and Pam(2)CGD	Mannose	DC	Enhanced responses upon targeting	[95]
i.v., s.c., i.m.	OVA-encoding mRNA	N/A	Passive	DC and macrophage	Enhanced responses upon i.v. immunization	[70]
i.v., s.c.	OVA peptide	Soluble poly IC and aCD40	GM3	CD169-expressing macrophages	Enhanced immune responses upon i.v. immunization, targeting and encapsulation of antigen	[54]
i.v.	Human papilloma virus peptide	CpG	Passive	cDC and pDC	Enhanced responses compared to soluble vaccine components	[96]
i.v.	OVA protein	Soluble poly IC and aCD40	GM3	CD169-expressing macrophages	Enhanced immune responses upon targeting and encapsulation of antigen	[83]
i.v.	OVA protein	α-Galactosylceramide	GM3	CD169-expressing macrophages	Enhanced CD8 T cell responses upon targeting	[97]

### 3.5. Adjuvant Encapsulation Augments Its Potency

In addition to antigen, encapsulation of adjuvant in liposomes can have a beneficial effect on the subsequent immune response (Table 2). For example, the TLR 9 agonist, CpG, was found to be more potent when present in the aqueous core of liposomes [87,89]. Indeed, administration of soluble CpG did not yield antitumor responses [92]. This finding was corroborated in a similar experimental setting, where liposomal, but not soluble, CpG could upregulate maturation markers on cDC, elicit IFN-y secretion by T cells, and induce potent antitumor responses [96]. In an alternative approach, Kwong and co-workers did not only encapsulate CpG in their liposomes, but also linked an activating anti CD40-agonistic antibody to the surface of the liposomes. Similar to previous observations, the potency of CpG-containing and anti-CD40-targeted liposomes was enhanced when compared to the soluble components. Indeed, upon immunization with these liposomes tumor outgrowth in mice was significantly delayed [90]. Thus, the potency of adjuvant is markedly increased upon liposomal encapsulation, which may be explained by enhanced delivery to the APCs. Hypothetically, the enhanced activation through liposomal delivery of TLR ligands is explained by the resemblance to the physiological route of activation (triggered by pathogens) as compared to administration of soluble adjuvant [89].

The use of liposomes also allows for incorporation of hydrophobic adjuvant in the bilayer, such as monophosphoryl lipid A (MPLA. Indeed, liposomal delivery of MPLA augmented cross-presentation by DCs, compared to soluble MPLA [98,99]. Furthermore, the use of liposomes also provides the opportunity to incorporate multiple TLR ligands as stimulus. When TLR 4 and TLR 7 were stimulated following combined liposomal delivery, synergistic effects were observed [100]. Similarly, this was also detected when ligands of TLR 3 and TLR 9 were combined in the same liposome, as co-delivery resulted in almost complete protection from a tumor challenge [88].

While the use of TLR ligands in (anti-cancer) vaccines is already well established, various other adjuvants, such as ligands for the cytosolic sensor STING, have been more recently included in liposomes for achieving cellular activation and subsequent anti-tumor responses. The potency of cyclic dinucleotides, which act as second messengers in the STING activation pathway was also found to be enhanced upon delivery in liposomes [93]. Delivery of cyclic dinucleotides encapsulated within the liposomal aqueous phase enhanced its cellular association and protection from tumor rechallenge as compared to free cyclic dinucleotides [101]. Also in the absence of an antigen, delivery of adjuvant-containing nanoparticles may prove beneficial to activate immune cells [102]. When this strategy was combined with irradiation to release tumor antigens, indeed, liposomal-encapsulated cyclic dinucleotides increased the anti-tumor immune response [91].

In summary, the potency of various adjuvants is enhanced when delivered by means of a liposomal carrier (Table 2).

## 4. Enhanced Delivery of Antigen to APCs Is a Third Component to Improve Cancer Vaccines

### 4.1. Different APCs for T Cell Priming

DCs are the designated cells to internalize and present antigen to prime naïve T cells. Importantly, cross-priming of CD8^+^ T cells occurs in secondary lymphoid organs and is dependent on both cellular cross-presentation capacity and the expression of costimulatory molecules [35]. While various cell populations appear to be capable of antigen presentation, cross-presentation by DCs is known to be superior to that by other APCs. However, DCs are a heterogeneous subset of cells with different capacities to (cross-)present antigens. DCs can be broadly divided in type 1 or 2 conventional, or plasmacytoid DCs (cDC1, cDC2, and pDC) [103]. In the mouse, DC1s are marked by XCR1 and Clec9a, whereas cDC2s can be identified by a lack of these markers, the expression of Sirpα and a higher expression of CD11b [103]. In addition, cDC1s in the murine spleen express CD8α, while migratory peripheral tissue cDC1s are marked by CD103 [25]. While both cDC1s and cDC2s are able to internalize and process antigen, cDC1s are most well known for their capacity to cross-present dead cells and to activate CD8^+^ T cells [104]. cDC1s have a lower proteolytic activity in their endosomal pathway, efficiently transfer peptides to the cytosol, and express relatively high levels of proteins related to MHC class I mediated presentation of antigen, which allows for efficient cross-presentation [28,105,106]. Indeed, in Batf3^-/-^ mice that lack cDC1s, an indispensable role for these cells in the initiation of an anti-tumor response was observed [107]. However, the type of antigen and environmental cues can dictate the extent to which various non-cDC1 DC subsets can cross-present antigen [108]. For example, pDCs, which are mostly well known for their capacity to produce type I IFN [103], gained the ability to cross-present antigen upon TLR stimulation [109,110]. In addition, cDC2s are mostly associated with antigen presentation in the context of MHC class II and activation of CD4^+^ T cells, but have been shown to be able to cross-present immune complexes and yeast particles [103,111,112]. In addition, recent work has shown that inflammatory cDC2s can also efficiently cross-present antigen [113].

The human counterparts of murine cDC1s and cDC2s are marked by the expression of XCR1, Clec9a, and BDCA3 versus BDCA1 (also known as CD141 and CD1c, respectively) [103]. In contrast to mice, cross-presentation in humans seems to be less restricted to cDC1s [114]. Indeed, human tissue-resident cDCs and pDCs were found to cross-present antigen in steady state. In addition, upon TLR stimulation blood-borne cDC1s and cDC2s were also able to cross-present [114]. Whether or not human DCs are equally potent to cross-prime T cells remains elusive [115]. Interestingly, the expression of Clec9a (DNGR-1) on cDC1s is evolutionarily conserved between mouse and human. DNGR-1 binds to freely accessible actin filaments that are present in dying cells [116]. Since this marker is exclusively expressed by cDC1s, this points to a major role for human cDC1s in the cross-presentation of antigen upon capture of dying cells [117,118,119]. Furthermore, in contrast to other human DC subsets, cDC1s express high levels of TLR 3 and stimulation of these cells with poly(I:C) induces potent maturation [120]. In the mouse, high expression of TLR 3 was determined to be important in the DNGR-1-mediated uptake and response to cell fragments that emerged from virally infected cells [121]. Therefore, the exclusive expression of DNGR-1 and TLR 3 in human cDC1s points toward an important role for these cells in uptake and subsequent cross-presentation of antigen derived from virally infected cells.

Recently, new human cDC subsets have been described that exceed the classical distinction between cDC1s and cDC2s. Especially within the classically defined CD1c-expressing cDC2 subset, more heterogeneity was observed [122]. For example, DC3 was identified as a DC subset that also expresses CD1c and therefore, sole discrimination of DC subsets on CD1c expression resulted in erroneous identification of DC3s as cDC2s. DC3 subset expresses a gene signature that is associated with acute and chronic inflammatory genes [123]. Interestingly, DC3s can be found in human tumors, can secrete high amounts of T cell polarizing cytokines, and are able to induce potent T cell responses [124,125]. In addition, two other subsets were described and defined as DC4 and DC5. Of these subsets, DC4 subset was not extensively described and is not marked by expression of either CD141 or CD1c, but does express certain markers also expressed by monocytes and may be a subset of non-classical monocytes [114,123]. DC5 subset is also referred to as Axl DC (AS DC), as it can be identified based on its expression of Axl, often in combination with Siglec-6 [123]. Since trajectory analysis showed that these cells can develop into other DC subtypes, these cells might be precursors and were also referred to as preDCs [126]. Here, we further refer to these cells as AS DCs. Interestingly, AS DCs can be found in the blood of healthy donors, but also in the blood of donors that suffer from a variety of cancers [127].

To summarize, cDC1s are designated as the major player in cross-priming, especially in mice. However, multiple DCs may play a role in cross-presentation and cross-priming, dependent on environmental cues. The recent identification of novel DC subsets, such as DC3s and AS DCs, opens new avenues in liposomal vaccination studies. Indeed, vaccination strategies may be tailored to optimally make use of the localization of DCs (e.g., blood-borne or tissue residency) as well as the capacity of DC subsets to cross-prime antigen [128].

### 4.2. Active Targeting Leads to Uptake by APCs of Choice

Although APCs are well known for their capacity to capture antigens, cancer vaccines may be functionalized to enhance the efficiency of uptake by specific types of APCs (Table 2). As mentioned, DCs (and especially cDC1s) may present as ideal targets for cancer vaccines, because of their excellent capacity to cross-present exogenous antigen [103]. In order to target DCs, Boks and co-workers decorated liposomes with Lewis X, which can act as ligand for the C-type lectin dendritic cell-specific intercellular adhesion molecule-3 grabbing non-integrin (DC-SIGN) on DCs and observed that surface coupling of targeting molecules enhanced liposomal binding and uptake by DC-SIGN-expressing target cells [99]. Additionally, mannose or a mannose-mimicking ligand can be used to target the mannose receptor (MR), to enhance liposome delivery to DCs [94]. Thomann and colleagues made use of this targeting strategy and prepared liposomes that contained ligands of TLR 1/2 or TLR 1/6 and a peptide antigen. Interestingly, additional incorporation of mannose to target DCs allowed lowering the dose of adjuvant used 100-fold, while maintaining similar anti-tumor effects [95]. Furthermore, Langerhans cells, APCs that are present in the skin, can be targeted via their expression of the C-type lectin receptor langerin. Modification of liposomes with one of its natural ligands, Lewis Y, or a glycomimetic ligand, resulted in enhanced uptake by Langerhans cells [129,130,131]. As an alternative target, multiple APCs expressed the receptor for phosphatidylserine (PS), which is exposed on dead, but not living, cells. Hence, inclusion of PS in the liposomal membrane can facilitate liposome uptake [91].

Next to the liposomal surface decoration with physiological ligands or a mimetic of these ligands for receptors on DCs, cell type specific antibodies can be conjugated to the surface of liposomes to specifically direct liposomes to the surface of a cell type of interest [132]. An alternative approach is to use the constant part of antibodies to target the Fc receptor on DCs [80,133].

While active targeting of liposomes can allow for tailored target cell delivery, it can also be used to influence subsequent intracellular antigen preservation and routing. Interestingly, antigen uptake via the MR or Fc receptors was shown to lead to endured colocalization of antigen with EEA1-positive early endosomal compartments, in sharp contrast to antigen that was taken up by scavenger receptors, which were quickly routed to lysosomes for degradation [33,34]. In contrast, uptake of antigen via DNGR-1 that is present on cDC1s was found to result in phagosome rupture and therefore efficient transfer of phagocytosed antigen to the cytosol for cross-presentation via the cytosolic route [31]. Thus, targeting of antigen-containing liposomes can facilitate enhanced presentation of the vaccine antigen to the immune system.

While we discussed that cDC1s, especially in mice but also in humans, appear to be highly relevant in cross-presentation of cell-associated antigen, to the best of our knowledge, liposomal targeting studies specifically directed to cDC1 markers, such as XCR1 and Clec9a, are still lacking. On a side note, cancer vaccine targeting to Clec9a was performed with antigen–antibody conjugates and antibody/antigen/poly (lactic-co-glycolic acid) (PLGA)nanoparticles and resulted in robust induction of antigen-specific T cell responses [134,135,136]. In conclusion, active targeting of liposomes by modification of their surface with targeting ligands can markedly increase the potency of vaccination.

### 4.3. Liposomal Vaccine Targeting to CD169-Expressing Cells in Mice and Men

As an alternative to DC targeting, recent studies investigated targeting to CD169-expressing splenic macrophages. These macrophages are strategically located in the spleen and are known to sequester blood-borne pathogens [137]. CD169-expressing macrophages recognize particulate antigens such as viruses and extracellular vesicles [138,139,140,141]. The CD169 receptor (also known as Siglec-1 or Sialoadhesin [142,143]) binds to α2,3-linked sialic acids that are present on glycoproteins or glycolipids, such as gangliosides [144]. Human immunodeficiency virus (HIV) buds of host lipid rafts are enriched in gangliosides. As a result, the viral envelope is decorated with ganglioside GM3 and indeed, these viruses are taken up by CD169-expressing cells [138,139,140]. In addition, artificial viral nanoparticles, which contained a gold core wrapped in a membrane exposing the physiological ligand for CD169, were taken up by CD169-expressing cells as an example of pathogen mimicry [145,146,147]. Next to the physiological ligand, decoration of liposomes with synthetic ligands for CD169 also resulted in efficient uptake by these cells [55,148]. Our group decorated liposomes with GM3 and detected robust targeting of liposomes to murine splenic CD169-expressing macrophages (Table 2) [54]. Of note, GM3-containing liposomes were minimally taken up by other cell types, for example, red pulp macrophages, that express low levels of CD169 [54]. Interestingly, CD169-expressing macrophages appear to closely collaborate with cDC1s upon liposomal immunization, where CD169-expressing macrophages capture and cDC1s cross-present liposomal antigen. Indeed, the presence of both cell types was essential in our liposomal vaccination strategy [83]. In addition, when liposomes were modified with α-Galactosylceramide (the natural ligand for invariant natural killer T (iNKT) cells), further enhancement of the liposomal efficacy for CD8^+^ T cell priming was observed [97]. Thus, liposome targeting to CD169-expressing splenic macrophages presents as an interesting strategy, as targeting is specific and upon liposome capture by CD169-expressing macrophages a potent immune response is elicited.

In addition to murine macrophages, human CD169-expressing splenic macrophages also bound liposomes that contained gangliosides [127]. Interestingly, in humans, CD169 is also constitutively expressed by the recently described AS DCs that are present in the blood [123]. As expected, AS DCs could also be targeted with ganglioside-containing liposomes. Upon delivery of antigen and adjuvant in liposomes, AS DCs presented peptide to CD8^+^ T cells and elicited IFN-y production by antigen-specific T cells [127].

Recently, the murine counterpart of AS DCs was termed transitional DC (tDC). Similar to AS DCs, tDCs were shown to express low levels of CD169 [149]. Since both CD169-expressing macrophages and tDCs are present in the spleen, it remains to be determined whether intravenously injected liposomes are taken up by tDCs next to CD169-expressing macrophages and whether they are involved in T cell priming [54].

**Figure 1:**
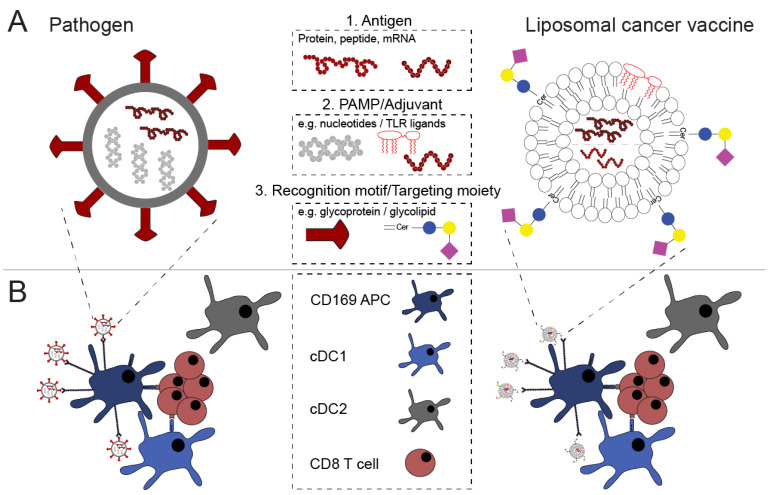
Pathogen mimicry of liposomal cancer vaccines augment their efficacy. (**A**) Pathogens harbor (1) antigenic proteins or peptide, from which an immune response can be elicited, (2) pathogen-associated molecular patterns (PAMPs) that activate the immune system, and (3) recognition molecules that allow for uptake by host cells. Liposomal cancer vaccines mimic pathogens by incorporation of (1) antigens, for example, protein/peptide, or mRNA, (2) TLR ligands or nucleotides to activate the immune system, and (3) modification of the liposomal surface with targeting moieties that allow for tailored interactions between the liposomal vaccine and immune cells. (**B**) Upon pathogen challenge or liposomal cancer vaccination, designated APCs take up and subsequently cross-present antigen in MHC class I to induce potent immune responses. Liposomal cancer vaccine mimicry can optimize vaccine interaction with the immune system that subsequently translates to robust antigen-specific immune responses.

Altogether, these studies using both mouse models and human cells demonstrated that targeting to CD169-expressing macrophages and DCs is an interesting approach to increase vaccine efficacy. Liposomes that contain components similar to those present in pathogens, such as gangliosides and nucleotides, can be used to target CD169-expressing APCs (Figure 1 and Table 2).

## 5. Conclusions and Future Perspectives

Recent studies have indicated that various liposomal characteristics as well as the route of immunization highly impact the subsequent immune responses and should be taken into account to improve liposomal cancer vaccines. Liposomes should be large and cationic in nature to yield depot formation from which antigens are slowly released, while smaller and anionic liposomes prove to be beneficial regarding their capability to enter the regional lymphatics to reach the LN. Recent studies using intravenous administration of liposomes or lipoplexes demonstrated the importance of an anionic nature of the particle (summarized in Table 1). Moreover, they indicated that this rather unconventional route of immunization in vaccination strategies can be more potent as compared to the more conventional immunization routes. While intravenous immunization is not feasible for vaccines that have to be delivered worldwide, such as COVID-19, this route may still be an option for cancer vaccines.

Numerous in vivo studies indicated that the immunogenicity and potency of delivered antigen and adjuvant, respectively, is markedly enhanced upon liposomal delivery. In addition, active targeting of liposomal cancer vaccines to professional APCs using ligands that that are also present in pathogens can further augment the potency of the immune responses (summarized in Table 2).

In our opinion, lessons learned from these preclinical studies in combination with our increasing knowledge of the immune system should be used for vaccine design in future (pre)clinical studies. Profiling of APC subsets revealed a previously underappreciated heterogeneity, in pathogen pattern recognition receptor-expression and also in the uptake of liposomal vaccines. Since liposomal surface decoration with targeting moieties can facilitate uptake by APCs of interest, the choice of vaccine adjuvant should also be based on the pathogen pattern recognition receptor-expression of the targeted APCs. In addition, recent identification of novel DC subsets (e.g., AS DCs) provides novel targeting strategies to be further explored.

In conclusion, the design of an optimal cancer vaccine should be inspired by the composition of pathogens in which antigen, adjuvant, and targeting moieties are present in a single particle, which may enhance the efficacy of liposomal cancer vaccines in the clinic.

## Data Availability

Not applicable.
